# Genital HSV-2 Infection Induces Short-Term NK Cell Memory

**DOI:** 10.1371/journal.pone.0032821

**Published:** 2012-03-22

**Authors:** Mohamed F. Abdul-Careem, Amanda J. Lee, Elishka A. Pek, Navkiran Gill, Amy E. Gillgrass, Marianne V. Chew, Sarah Reid, Ali A. Ashkar

**Affiliations:** McMaster Immunology Research Centre and Institute for Infectious Diseases Research, Department of Pathology and Molecular Medicine, McMaster University Health Sciences Center, McMaster University, Hamilton, Ontario, Canada; University of London, St George's, United Kingdom

## Abstract

NK cells are known as innate immune cells that lack immunological memory. Recently, it has been shown that NK cells remember encounters with chemical haptens that induce contact hypersensitivity and cytomegalovirus infection. Here, we show the existence of NK cell memory following HSV-2 infection. Stimulation with HSV-2 Ags led to higher IFNγ production in NK cells that were exposed 30 days previously to HSV-2, compared to NK cells from naïve mice. More importantly, this increased production of IFNγ in NK cells was independent of B- and T- lymphocytes and specific for the HSV-2 Ags. We also showed that previously exposed NK cells in a B- and T-lymphocyte free environment mediate protection against HSV-2 infection and they are necessary for the protection of mice against HSV-2 infection. Collectively, NK cells remember prior HSV-2 encounters independent of B- and T- lymphocytes leading to protection against HSV-2 mediated morbidity and mortality upon re-exposure.

## Introduction

NK cells are known as innate immune cells and mediate cytotoxicity against cells infected with pathogens early following infection [1]. NK cells are also a source of cytokines such as IFNγ and TNFα and are therefore instrumental in activating the adaptive arm of the immune system. Although NK cells possess various activating and inhibitory receptors, they lack classical antigen (Ag) recognizing receptors. Consequently, NK cells are unable to mount antigen specific immune responses, expand in response to Ags and display immunological memory. A few recent records have challenged the notion that NK cells lack the capability to elicit antigen specific immune responses that may lead to the host acquiring NK cell memory [2]–[6].

Existence of memory in the invertebrate innate immune system has been shown using a copepod parasite infection model [7]. Furthermore, recent records have indicated the existence of a subset of NK cells that remember activation by cytokines [8], encounters with chemical haptens [9] and mouse cytomegalovirus (MCMV) infection [10]. These subsets of NK cells were phenotypically similar to naïve NK cells, but distinct because they lack constitutive expression of IFNγ and granzyme B. However, this subset of NK cells could be activated to produce higher IFNγ and kill target cells like naïve NK cells [8], [10]. Using a mouse model of MCMV infection, Sun et al. [10] identified a subset of memory cells as Ly49H+ NK cells. These Ly49H+ NK cells could undergo expansion, contraction, memory maintenance and secondary recall response following recognition of the MCMV protein m157. However, the activity of these memory NK cells was not against other viral or chemical antigens [9], [10].

The duration of memory response displayed by NK cells appears to be varied. Memory response following the application of chemical haptens appears to persist for about a month [9], whereas that recorded against MCMV persisted for several months [10]. It has also been observed that NK cells that are activated by DCs provide protection up to one year against B16 melanoma in a mouse model [11]. Interestingly, this long-term protection mediated by the NK cells following DC treatment relied on CD4+ T cells and was abrogated following elimination of IFNγ. In agreement with the findings of the later study, following human immunization against rabies, NK cells acquired the capability of higher IFNγ production and degranulation upon re-exposure for up to 4 months [12]. This long term Ag specific proliferation and enhanced NK cell activity has been shown to be dependent on IL-2 signaling from memory CD4+ T cells [12].

A high percentage of NK cells possess the Ly49H+ receptor that specifically recognizes MCMV Ag, m157 [13], which facilitated the characterization of memory NK cells generated against MCMV [10]. It is not known whether NK cells can display memory responses against any other viral infections and if they do, whether it is B- and T- lymphocyte independent since it has been shown that NK cell mediated protection induced by DCs is dependent on CD4+ T cells [10] and increased proliferation and activity in rabies Ag re-exposed human NK cells depend on IL-2 signaling derived from memory CD4 T-cells [12]. Here, we first investigated whether NK cells are able to remember and respond following re-exposure to another viral infection other than MCMV. We then examined if this can occur in the absence of T- and B-lymphocytes, For this end, we used a well-established mouse model of genital herpes simplex virus type-2 (HSV-2) infection in which it is known that NK cells are important for protection [14]. We found that, unlike naïve NK cells, the NK cells that encountered HSV-2 previously were capable of a higher IFNγ response upon re-exposure to HSV-2 Ags, but not to other stimulants such as Poly I:C. *Ex vivo* stimulation of HSV-2 exposed NK cells in a B- and T-lymphocyte free environment could also increase IFNγ production when compared to NK cells from naïve mice. We also showed that previously exposed NK cells were essential to confer significant protection against secondary HSV-2 infection induced morbidity and mortality and this protection was specific for HSV-2 infection.

## Materials and Methods

### Ethics

All animal experiments were approved by the Animal Research Ethics Board (AREB) of McMaster University under AUP number: 10-02-12.

### Mice

C57BL/6 (Charles River Laboratory, Quebec, Canada) were purchased and RAG1^−/−^ (Taconic, Germantown, N.Y.) mice were bred and maintained at the Central Animal Facility, University of McMaster, Hamilton, Ontario, Canada. All the experiments were conducted according to guidelines laid by the institutional animal care committee.

### HSV-2 infection and immunization

Sexually mature (6–8 weeks) mice in diestrus (treated with long acting progesterone preparation at 2 mg per mouse five days before) were immunized intra-vaginally or intra-nasally with either 1×10^5^ (C57BL/6) or 1×10^4^ PFU (RAG1^−/−^) thymidine kinase-deficient (TK^−/−^) HSV-2. RAG1^−/−^ and C57BL/6 mice that were in diestrus were infected with five times the lethal dose (5×10^3^ PFU, RAG1−/−) or 1×10^5^ PFU (C57BL/6) of wild type HSV-2 strain 333 intra-vaginally 21 days post immunization (dpi) [14]. Mice were observed for HSV-2 lesions and the end point daily for 21days post-infection as has been described previously [15]. Briefly, a score of 0, 1, 2, 3, 4 and 5 were assigned respectively for no lesions, slight reddening of external genitalia, swelling and redness of external genitalia, severe swelling plus hair loss around external genitalia, ulceration associated with swelling and hair loss around external genitalia, and extended ulceration plus hind quarter paralysis. When the mice reached the scale of five, the mice were euthanized according to institutional animal care guidelines.

### NK cell depletion

Female RAG1^−/−^ mice were injected intraperitoneally with 200 µg of anti-mouse NK1.1 antibody (PK136 mouse immunoglobulin G2a hybridoma HB191; ATCC) on day −2 and −1 and challenged on day 0 with 5×10^3^ PFU of wild type HSV-2. NK1.1^+^-cell depletion efficiency was confirmed at the time of HSV-2 infection using a sub set of mice. Following the infection on day 0, two more injections were given intraperitoneally on day +2, and +5 and every 3–4 days thereafter if necessary.

### B16F10 tumor challenge

B16F10 cells were cultured under normal culture conditions in α-MEM supplemented with 10% (v/v) FBS, 1% (v/v) L-glutamine, 1% (v/v) penicillin-streptomycin, 1% (v/v) Hepes (Gibco, Grand Island, NY, USA) and 0.001% 2-mercaptoethanol (2ME). C57BL/6 mice immunized with TK^−/−^ HSV-2 3 weeks prior to i.v. injection with 1×10^6^ B16F10 cells that were in the log phase. Two weeks following the challenge lungs were excised and fixed in 2% paraformaldehyde for 48 hours, and tumor nodules were counted using a stereoscope.

### Vaginal Virus titration

Vaginal washes were collected 1–3 days post-infection by pipetting 2×30 µl PBS in and out 6–8 times. A plaque assay was performed for the titration of HSV-2 virus in vaginal washes which has been described previously [16]. Briefly, confluent monolayers of Vero cells were grown in 12 well plates using α-MEM (Gibco Laboratories, Burlington, Canada) supplemented with 2 mmol/l L-glutamine, 1% penicillin-streptomycin and 5% heat inactivated fetal bovine serum. Serially diluted vaginal wash samples were added to the monolayer and incubated at 37°C with 5% CO^2^. Following one hour of incubation, the plates were added with an overlay of α-MEM supplemented with 0.05% human immune serum (Canadian Blood Services). The plates were incubated again for 48 hours and then fixed and stained with crystal violet before counting the plaques under an inverted microscope.

### Preparation of HSV-2 and control cell lysate

The HSV-2 lysate was prepared using HSV-2 cultured on Vero cells (1×10^9^ PFU) following UV inactivation and 3× freeze-thaw cycles, The efficiency of lysis, as measured by the absence of live virus, was tested on Vero cells. Uninfected cells were also subjected UV inactivation and freeze-thaw cycles in order to prepare the control cell lysate. Following the lysis, the protein concentration was measured using a Bradford assay (Bio-Rad) according to the manufacturer's instructions.

### *Ex vivo* stimulation of NK cells and intracellular cytokine staining

Splenocytes were isolated as a single cell suspension using standard methods and 2×10^6^ cells per well were cultured in complete medium (RPM1 medium 1640 supplemented with 2 mmol/l L-glutamine, 1% penicillin-streptomycin and 10% heat inactivated fetal bovine serum) with either HSV-2 lysate (10 µg/ml), Vero cell lysate (10 µg/ml), poly I:C (10 µg/ml) or RPM1 medium for 18–21 hours [17] in 96 well plate. Protein transport inhibitor, GolgiStop (BD Biosciences), was added to the culture after 12 hours. At the end of stimulation non-specific antibody binding was blocked with anti mouse CD16/32 Fc blocker (eBioscience). Cell surface staining was done using anti mouse NK1.1-phycoerythrin (eBioscience), and anti mouse CD3-alexa flour 700 (Biolegend). The cells were fixed and permeabilized using cytofix/cytoperm buffer (BD Biosciences) before staining with anti mouse IFNγ-APC antibody. Flow cytometry data were collected on the FACSCanto (BD Biosciences) and analyzed using FlowJo version 7.2.5.

### *Ex vivo* extracellular NK cell staining

Splenocytes and lymph nodes were isolated as a single cell suspension using standard methods and 1×10∧6 cells per well plated in a 96 well plate. Non-specific antibody binding was blocked with anti mouse CD16/32 Fc blocker (eBioscience). Cell surface staining was done using anti mouse NK1.1-phycoerythrin (eBioscience) and anti mouse CD-3-alexa flour 700 (Biolegend) for lymph nodes as well as anti mouse CD27-PerCP (eBioscience) and anti mouse CD11b-Pe-Cy7 (eBioscience) for splenocytes.

### ELISA

Culture supernatants collected at the end of the ex vivo stimulation were assayed for IFNγ production by the DuoSet ELISA kit (R&D Systems, Minneapolis, MN). The plates were read using the Saphire ELISA plate reader at 450 nm wavelength.

### Data analysis

All data except survival proportions were analyzed by Student's *t*-test to identify differences between groups using the statistical package, MINITAB® release 14 (Minitab Inc., State College, Pennsylvania, USA). Differences in survival between TK^−/−^ HSV-2 immunized and unimmunized control as well as between NK cell depleted and control TK^−/−^ HSV-2 immunized mice were analyzed by χ^2^ test using GraphPad Prism 4 (GraphPad Prism Software, La Jolla, CA). Comparisons were considered significant at *P*≤0.05.

## Results

### Prior exposure of NK cells to HSV- 2 increases IFNγ production upon re-exposure

**Figure 1:**
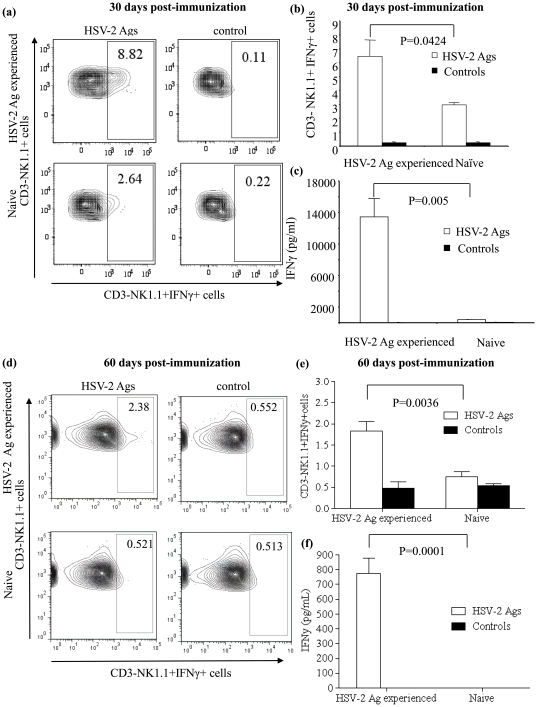
Prior exposure of NK cells to HSV- 2 Ags increases IFNγ production upon re-exposure. Six to eight weeks old female C57BL/6 mice in diestrus were immunized intra-vaginally with 1×10^5^ PFU per animal of TK^−/−^ HSV-2 (n = 3–5 per group). Thirty and sixty days following immunization, splenocytes were isolated and stimulated with HSV-2 lysate for 18–21 hours. The resultant culture supernatants were collected to assay IFNγ concentration by ELISA and the NK cells were stained for intracellular IFNγ production. (a and d): representative FACS plots showing CD3-NK1.1+IFN-γ+ cells in the spleens of HSV-2 exposed and naive mice, (b and e): percentage of CD3-NK1.1+IFN-γ+ cells in the spleen of HSV-2 exposed and naive mice, (c and f) IFN-γ production in HSV-2 exposed and naive splenocytes upon stimulation with HSV-2 Ags or control cell lysate.

It has been shown that immunization with the TK^−/−^ strain of HSV-2 protects C57BL/6 mice from wild type HSV-2 infection associated morbidity and mortality [18]. The protection mediated by immunization with the TK^−/−^ HSV-2 strain has been related to T cells and production of IFNγ [19]. Since NK cells also play a critical role in innate protection against HSV-2 infection [14] and could be a source of IFNγ [18], we investigated whether previously exposed NK cells produce more IFNγ upon re-exposure to HSV-2 Ags *in vitro*. For this purpose, female C57BL/6 mice were immunized with TK^−/−^ HSV-2 alongside unimmunized controls. Thirty and sixty days following immunization, the harvested splenocytes were stimulated with HSV-2 lysate and the total IFNγ production in culture supernatants was determined. An increase in IFNγ concentration in the culture supernatants collected at the end of the stimulation periods (30 days, P = 0.005; Fig. 1c and 60 days, P = 0.0001; Fig. 1f) was observed in splenocytes of the immunized mice when compared to the unimmunized controls. Since the cellular source of IFNγ in the culture supernatants is not clear, we also performed IFNγ intracellular cytokine staining for NK cells. NK cells from the TK^−/−^ HSV-2 immunized mice showed increased intracellular IFNγ production when compared to that observed in unimmunized controls 30 days (P = 0.0424; Fig. 1a and b) and 60 days (P = 0.0036; Fig. 1d and 1e) post-immunization. The increased *ex vivo* stimulation induced IFNγ production observed in NK cells that encountered HSV-2 Ags previously (30 and 60 prior) when compared to naive NK cells potentially reflects immunological memory in NK cells (8–10). In comparison to the 30 day post-immunization time point, there is a noticeable decrease in the percentage of NK1.1+IFNγ+ NK cells at 60 day post-immunization. This is similar to a normal adaptive immune response, in which the number of adaptive cells within the local environment decreases proportionally with time as the infection abates and memory is developed. However, this set of data does not provide evidence that the observed increase in IFNγ concentration in HSV-2 experienced NK cells is due to NK cell memory and is independent of B- and T-lymphocytes in the culture environment.

### Prior exposure to HSV-2 increases IFNγ production in NK cells in the absence of B- and T- lymphocytes following re-exposure

**Figure 2:**
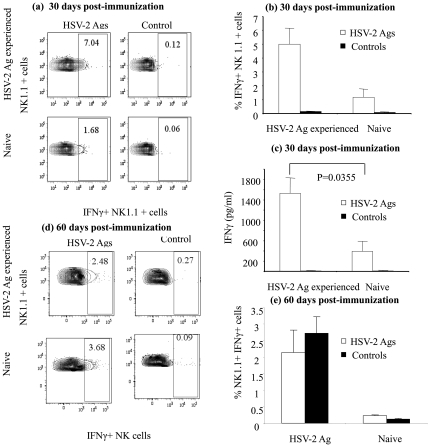
Prior exposure to HSV-2 Ags increases IFNγ production in NK cells even in the absence of B- and T- lymphocytes. Female RAG1^−/−^ mice were immunized intra-vaginally with 1×10^4^ PFU per animal TK^−/−^ HSV-2 (n = 3–5). Thirty or sixty days following immunization, splenocytes were isolated and stimulated with HSV-2 lysate for 18–21 hours. The resultant culture supernatants were collected for the determination of IFNγ concentration by ELISA and the NK cells were stained for intracellular IFNγ production. (a and d): representative FACS plots showing CD3-NK1.1+IFN-γ+ cells in the spleens of HSV-2 exposed and naive mice, (b and e): percentage of CD3-NK1.1+IFN-γ+ cells in the spleens of HSV-2 exposed and naive mice, (c) IFN-γ production in HSV-2 exposed and naïve splenocytes 30 days post-immunization following stimulation with HSV-2 or control cell lysate. The experiment was repeated twice with similar results.

It has been suggested that T cells, particularly CD4+ T cells, can induce long term NK cell reactivity to antigens [11]. However, recently it has been shown that NK cell memory against hapten is mediated independent of B- and T-lymphocytes [9]. It is not well defined if viral infections can induce NK cell memory in B- and T- lymphocyte deficient mice. To determine whether NK cell memory of HSV-2 is also mediated independent of B- and T- lymphocytes, we used RAG1^−/−^ mice, which provide a mature B- and T- cell free environment [20] for the subsequent experiments. We extended the above observation by conducting the same experiments in RAG1^−/−^ mice. We saw significant intracellular IFNγ production in NK cells and higher IFNγ production in splenocyte culture supernatants in the RAG1^−/−^ mice that had experienced HSV-2 30 days prior (P = 0.0355, Fig. 2 a–c) when compared to the naïve controls exposed to the HSV-2 Ags. We then examined if NK cells could have long-term memory of HSV-2 infection. The RAG1^−/−^ mice were immunized with TK^−/−^ HSV-2 and 60 dpi, NK cell activity against HSV-2 was tested. In this case we found that the concentration of IFNγ in the culture supernatants (data not shown) as well as in the intracellular environment in previously exposed NK cells were not different from that of naïve NK cells upon re-exposure to HSV-2 Ags (P = >0.05; Fig. 2 d and f).

### NK cells that experienced HSV-2 provide protection against subsequent HSV-2 challenge

**Figure 3:**
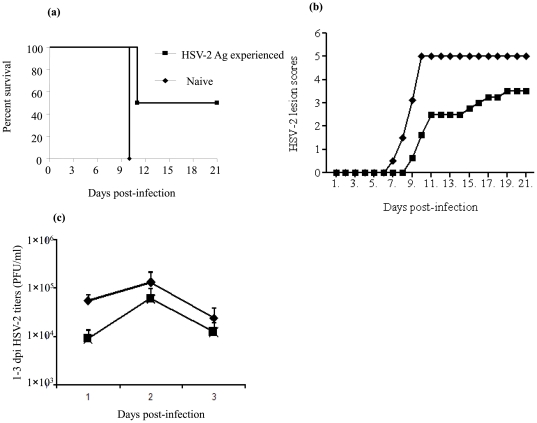
NK cells previously exposed to HSV-2 Ags reduce the consequence of secondary HSV-2 infection in mice that lack B- and T- lymphocytes. A group of female RAG1^−/−^ mice (n = 4–5) were immunized intra-vaginally with TK^−/−^ HSV-2 (1×10^4^ PFU per animal) alongside unimmunized controls. Both groups were infected 3 weeks following immunization with a lethal dose of wild type HSV-2 (5×10^3^ PFU per animal) intra-vaginally. The mice were observed for genital lesions and vaginal washes were collected 1–3 days post-infection. (a), (b) and (c) illustrate the percentage survival, the vaginal lesion scores and viral titers in vaginal washes. The experiment was repeated twice with similar results.

Since previously exposed NK cells were capable of producing a higher concentration of IFNγ in the absence of mature B- and T- lymphocytes upon re-exposure to HSV-2 Ags, we investigated whether these NK cells could reduce HSV-2 induced morbidity and mortality upon re-exposure. Indeed, protection attributable to IFNγ production and NK cells in the presence of B- and T-lymphocyte has been recorded [19], [20]. For this experiment, RAG1^−/−^ mice devoid of B- and T-lymphocytes were immunized with TK^−/−^ HSV-2 intra-vaginally and then infected with a lethal dose of (5×10^3^ PFU) wild type HSV-2 intra-vaginally three weeks following immunization. A set of unimmunized mice was challenged as a control. Previously exposed and naïve mice developed genital HSV-2 pathology, but the onset was delayed and there was decreased lesion severity in mice that had encountered HSV-2 previously (Fig. 3b). Only 50% of previously exposed mice reached end point in the immunized group, whereas all the naive mice reached end point (P = 0.0082; Fig. 3a). The difference in the lesion scores and survival between previously exposed and naïve mice were reflected in the vaginal HSV-2 titers being marginally lower in the mice that had encountered HSV-2 previously (Fig. 3c).

### Response of NK cells that experienced HSV-2 was specific for HSV-2

**Figure 4:**
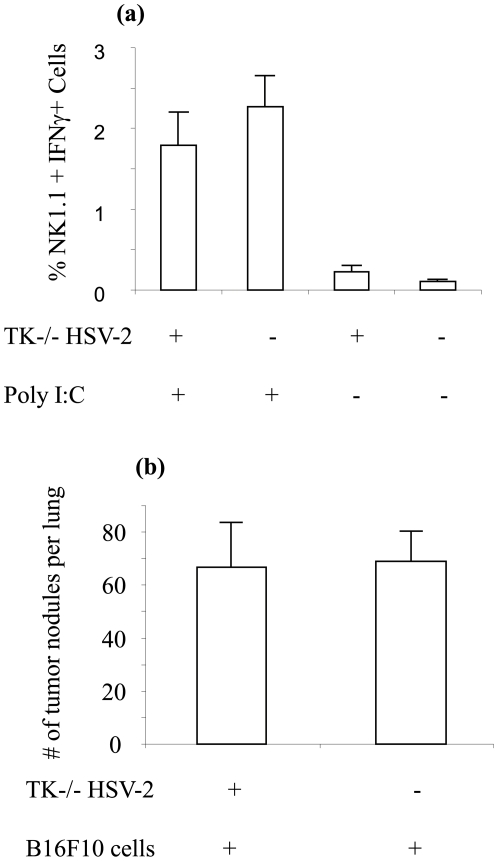
Response of NK cells that experienced HSV-2 was specific for HSV-2. (a) Six to eight weeks old female C57BL/6 mice in diestrus were immunized intra-vaginally with 1×10^5^ PFU per animal of TK^−/−^ HSV-2 (n = 5) alongside unimmunized controls (n = 5). Thirty days following immunization, splenocytes were isolated and stimulated with poly I:C (10 µg/ml) for 18–21 hours. The NK cells were stained for intracellular IFNγ production and percentage of CD3-NK1.1+IFN-γ+ cells in the spleen of HSV-2 exposed and naive mice are presented. (b) Six to eight weeks old female C57BL/6 mice in diestrus were immunized intra-vaginally with 1×10^5^ PFU per animal of TK^−/−^ HSV-2 (n = 9). Three weeks following immunization, the mice were challenged with B16F10 cells (1×10^6^ cells/mouse) i.v. alongside unimmunized controls (n = 9). Counts of tumor nodules in the lung two weeks following the tumor challenge are presented.

It has been shown that antigen presenting cells such as DCs can activate NK cells up to one year, which could elicit non-specific responses protecting against a number of tumors [11]. Thus we investigated whether the NK cell responses we observed in vivo and ex vivo were Ag specific. For this purpose C57BL/6 female mice were immunized with TK^−/−^ HSV-2 and splenocytes were collected 30 dpi and stimulated with synthetic double stranded RNA (poly I:C) with unimmunized control splenocytes. Intracellular IFNγ responses between HSV-2 Ag experienced and control NK cells were similar (Fig. 4a; P>0.05). This observation suggested that the significant NK cell IFNγ response observed when re-stimulated with HSV-2 Ags was specific for HSV-2 Ags. We also investigated whether the heightened and protective NK cell response observed in TK^−/−^ HSV-2 immunized mice, could protect against other Ags such as tumor Ags. For this end, we challenged the TK^−/−^ HSV-2 immunized mice with B16F10 cells 3 weeks following immunization. The number of tumor nodules in the lung of TK^−/−^ HSV-2 immunized mice was not different from that observed in unimmunized control mice (Fig. 4b; P>0.05).

### *In vivo* depletion of NK cells abrogates protection against HSV-2 in immunized RAG-1^−/−^ mice but not in immunized wild-type mice

**Figure 5:**
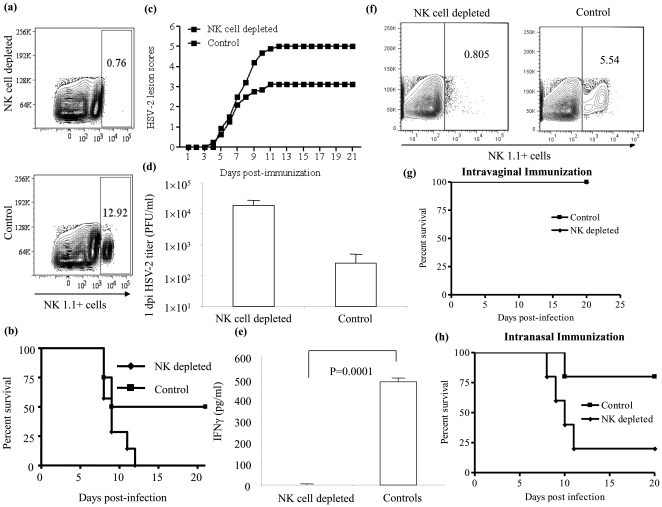
Depletion of previously exposed NK cells affects the B- and T-lymphocyte independent but not dependent protection against HSV-2 infection. A group of female RAG1^−/−^ (n = 4–5 per group) and C57BL/6 mice (n = 4 per group) were immunized intra-vaginally or intra-nasally with TK^−/−^ HSV-2 (1×10^4^ PFU and 1×10^5^ PFU per animal respectively). One of the groups of RAG1^−/−^ and C57BL/6 mice, received anti NK1.1 antibody (200 µg) on day −2, −1, +2 and +5. NK cell depleted and undepleted control groups were infected 3 weeks following immunization (day 0) with a lethal dose of wild type HSV-2 (5×10^3^ PFU or 1×10^5^ PFU per animal respectively) intra-vaginally. The mice were observed for genital lesions (all groups) and vaginal washes were collected 1–3 days post-infection for RAG1^−/−^ mice. (a) representative FACS plots showing the efficiency of NK cell depletion, (b), (c), (d) and (e) illustrates the percentage survival, the vaginal lesion scores, viral titers and concentration of IFN-γ in vaginal washes of RAG1^−/−^ mice. (f) representative FACS plots showing the efficiency of NK cell depletion, (g) and (h) illustrates the percentage survival of C57BL/6 mice immunized intra-vaginally and intra-nasally, respectively. NK cell depleted and control C57BL/6 mice immunized intra-vaginally were free of any vaginal pathology (data not shown).

Although RAG1^−/−^ mice are free of mature B- and T- lymphocytes, a group of TK^−/−^ HSV-2 immunized RAG1−/− mice were depleted of NK cells using anti-NK1.1 antibody 3 weeks post-immunization (Fig. 5a) and then infected with the lethal dose (5×10^3^ PFU per animal) of wild type HSV-2 to rule out the possible contribution of other leukocytes, such as macrophages and DCs, for the protection observed in immunized mice, The NK cell depletion (Fig. 5f) experiment was repeated with C57BL/6 mice with appropriate immunization and challenge doses to ascertain the role of NK cells in the presence of T and B cells in the mice. As illustrated in Fig. 5b–e and Fig. 5g respectively, depletion of previously exposed NK cells abolished the significant protection mediated by exposed NK cells in RAG1^−/−^ (50% vs 0%) but not in the wild type mice. This lack of protection in RAG1^−/−^ was reflected in higher HSV-2 titers, higher lesion scores, as well as in the undetectable IFN-γ in the vaginal washes of the NK cell depleted group of RAG1^−/−^ mice. Hence, previously exposed NK cells, in the absence of B- and T- cells, are necessary for the protection against the consequences of HSV-2 infection. While intra-vaginally immunized wild type mice were protected from lethal HSV-2 infection in the absence of NK cells (Fig. 5g), previously exposed NK cells appear to be necessary for protection in intra-nasal immunization where there was only a 20% survival rate in mice that were depleted of NK cells compared to 80% in the immunized control group (Fig. 5h). Overall, previously experienced NK cells are necessary for protection against vaginal HSV-2 infection in the absence of B- and T-cells as well as in the context of intra-nasal immunization.

### HSV-2 Ag experienced and naive NK cells are phenotypically similar

**Figure 6:**
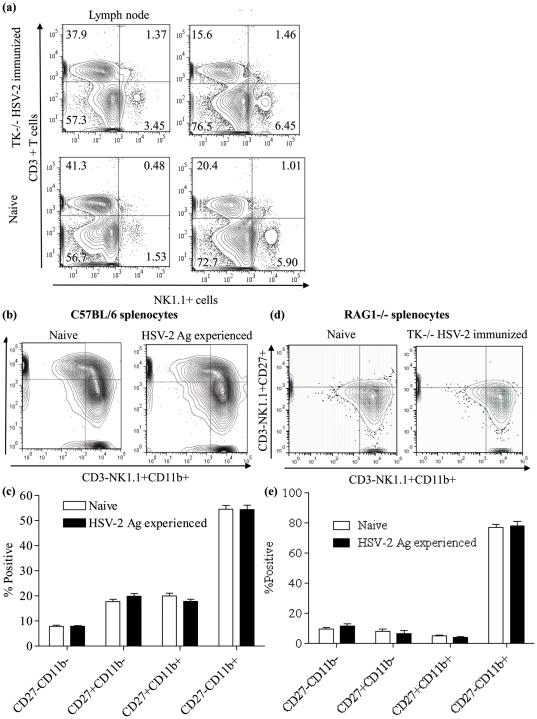
HSV-2 Ag experienced and naive NK cells are phenotypically similar. Six to eight week old female C57BL/6 (n = 3–5) and RAG1−/− mice (n = 4–6) in diestrus were immunized intravaginally with 1×10^5^ PFU per animal of TK−/− HSV-2 alongside unimmunized controls. Two days post-immunization cells (lymph node and spleen) from three C57BL/6 immunized and three C57BL/6 control mice were isolated and stained to compare the CD3-NK1.1+ cell numbers in immunized and control mice. Thirty days following immunization, C57BL/6 and RAG1−/− splenocytes were isolated from immunized (n = 5) and unimmunized controls (n = 5) and stained to analyze NK cell phenotype via CD27 and CD11b. (a) representative FACS plots showing CD3-NK1.1+ cells in the lymph node and spleen of HSV-2 exposed and naive mice 2 days following immunization, (b and d) representative FACS plots showing CD3-NK1.1+ cells in the spleens of HSV-2 exposed and naive mice on a CD27 and CD11b cross-gate, (c and e) percentage of CD27−CD11b+, CD27+CD11b−, CD27+CD11b+, and CD27−CD11b+ NK cells in HSV-2 exposed and naive mice.

It has been shown that HSV-2 infection in mice increase the NK cell numbers in spleen, lymph nodes and nervous tissues [21]. To elucidate the NK cell expansion following TK^/−^ HSV-2 immunization, spleen and lymph node NK cell numbers in wild type mice were observed 2 days following immunization. The average NK cell numbers in TK^/−^ HSV-2 immunized lymph node (2.67% vs 1.54%) and spleen (6.47% vs 5.49%) were higher when compared to the controls (Fig. 6a). To elucidate whether the increased NK cells in TK^/−^ HSV-2 immunized mice are phenotypically different, we characterized the NK cells in immunized and control mice using CD27 and CD11b markers 30 days post-immunization. It has been previously shown that the combination of CD27 and CD11b can portray NK cell phenotype and maturation status [22]. As has been illustrated in Fig. 6b–e, CD27−CD11b+, CD27+CD11b−, CD27+CD11b+, and CD27−CD11b+ NK cells in HSV-2 exposed and naive mice were not different (P>0.05).

## Discussion

It has been shown that NK cells can remember past encounters of chemical haptens, activation by cytokines and MCMV Ag, m157 [8]–[10]. In this study we show the existence of memory against another viral infection, HSV-2. NK cells that experienced viral Ags by means of immunization with TK^−/−^ HSV-2 had a higher potential of producing IFNγ independent of B- and T- lymphocytes when compared to naïve NK cells. The elicited NK cell IFNγ response was specific to HSV-2, which is necessary to control HSV-2 induced morbidity and mortality in RAG1^−/−^ mice that are deficient of B- and T- lymphocytes.

Cooper and co-workers [8] isolated NK cells from the spleen of RAG1^−/−^ mice, activated those NK cells by exposing the cells to IL-12 and IL-15 overnight, adaptively transferred and showed that transferred activated NK cells proliferate better and had acquired capability of eliciting a higher IFNγ response up to 3 weeks following adaptive transfer when compared to that elicited by naïve NK cells. Similarly, we observed that the NK cells that experienced HSV-2 Ags previously had a higher IFNγ response upon re-stimulation with HSV-2 Ags 30 days but not 60 days following the original exposure. The finding of Cooper and co-workers and our study show that [1] previously activated or Ag experienced NK cells are capable of eliciting higher IFNγ responses upon re-activation or re-exposure to the same Ag and [2] the NK cell remembrance of previous encounters of activation by cytokines or HSV-2, in the absence of B- and T- lymphocytes, is short term. The observation of short term NK cell memory is in agreement with the observations made in another study conducted using chemical haptens where the authors have observed that NK cell, independent of B- and T-lymphocytes can mediate hypersensitivity following the application of chemical haptens such as 2,4-dinitrofluorobenzene and oxazolone up to 4 weeks [9]. However, Sun et al. [10] observed the existence of memory NK cells generated against MCMV for at least 10 weeks. The discrepancy in results between our study and that described by Sun et al. [10] may be due to the differences in mice (RAG1^−/−^ vs C57BL/6) and virus (HSV-2 vs MCMV) used for the experiments.

Another important finding emerging from our study is that the higher capability of IFNγ response of previously HSV-2 encountered NK cells was specific for the encountered Ags. In agreement with this *in vitro* data, we also observed that the previously HSV-2 encountered RAG1^−/−^ mice were protected against wild-type HSV-2 challenge, but not against B6F10 melanoma challenge. Altogether, this set of observations suggests that the heightened response in re-exposed NK cells that were previously experienced with HSV-2 Ags was a specific response and agrees with the observation made by O'Leary et al. [9]. The later study used three different haptens and showed that the short-term memory expressed by NK cells was specific for the hapten used.

We also observed that previously HSV-2 encountered NK cells were necessary to control wild-type HSV-2 induced morbidity and mortality in a B- and T- lymphocyte free environment. Our results are in agreement with the data of Sun et al. [10] who have observed that the transfer of MCMV specific memory NK cells to new born mice could elicit a protective response (75%) against lethal MCMV infection when compared to the transfer of an equal number of naïve NK cells (20%). However, in intra-vaginally immunized wild type mice, the presence of B- and particularly T-cells was able to provide protection and induce the production of IFNγ in the absence of NK cells. It has been shown that early NK cell IFNγ production is important in controlling HSV-2 viral infection [14]. Without this early IFNγ production, naïve mice infected with HSV-2 become more susceptible to the infection [14]. However, intra-vaginal immunization with TK- HSV-2 is a potent immunizer in which intra-vaginal associated lymphoid tissues (iVALT) develop and house a number of immune cells, including CD4+ T-cells within the vaginal tissue [23]. It is likely that these previously exposed antigen specific CD4+ T-cells were able to quickly respond and produce IFNγ in response to vaginal HSV-2 infection in the place of early NK cell derived IFNγ production. In intranasal immunization, which does not develop a strong local immune response when compared to intra-vaginal immunization, depletion of NK cells reduced protection in immunized mice. In this case, previously experienced NK cells are crucial in providing protection against HSV-2 challenge. Our data and previous findings therefore reveal that Ag experienced NK cells can clearly play a protective role against infections caused by viruses such as HSV-2 and MCMV and this could be exploited in potential vaccination regimes against these viral infections.

Overall, this study provides evidence that NK cells can remember past encounters with HSV-2 Ags and this short-term memory is independent of B- and T- lymphocytes. Our study extends previous observations that recorded NK cell memory of a unique murine viral infection, MCMV, to another viral Infection, HSV-2. However, we observed a short-term (30 days) but not a long-term (60 day and more) memory of HSV-2 infection in an environment free of B- and T-lymphocytes. In agreement with these findings, we observed that the HSV-2 antigen experienced NK cells show a heightened IFNγ response for an extended period in the presence of the adaptive arm of the immune system (up to 60 days) than in the absence of T and B cells (up to 30 days). This finding suggests that cytokines may be playing a role in the activation of NK cells. The previous observations and the current findings suggest that the length of remembrance of Ags by NK cells vary depending on the encountered Ags or possibly the presence of T cells in the environment. The latter view could be supported by the finding that human NK cells can proliferate and increase the activity in an Ag specific manner for several months against rabies viral Ags with helper signals from memory CD4 cells and accessory cells such as macrophages [12]. Further, NK cells activated by DC treatment can elicit protective responses against melanoma challenge up to one year with helper signal from CD4+ T cells [11]. Although there was an increase in the NK cells in the secondary lymphoid organs early following immunization against HSV-2, the HSV-2 Ags experienced NK cells were phenotypically similar to the naïve NK cells. Importantly, the elicited memory for HSV-2 Ags was specific for the encountered Ags. Due to the lack of knowledge on the NK cell receptor used for the recognition of HSV-2 Ags, the mechanism and the subset of NK cells responsible for remembrance of HSV-2 Ags were investigated. It is important to note that MCMV is unique as this is currently the only virus which NK cells have a specific receptor, Ly49H [13].
